# Production and characterization of a novel long-acting Herceptin-targeted nanobubble contrast agent specific for Her-2-positive breast cancers

**DOI:** 10.1007/s12282-014-0581-8

**Published:** 2015-02-19

**Authors:** Qiongchao Jiang, Shaoyun Hao, Xiaoyun Xiao, Jiyi Yao, Bing Ou, Zizhuo Zhao, Fengtao Liu, Xin Pan, Baoming Luo, Hui Zhi

**Affiliations:** 1 Department of Ultrasound, Sun Yat-sen Memorial Hospital, Sun Yat-sen University, Guangzhou, Guangdong People’s Republic of China; 2 Key Laboratory of Malignant Tumor Gene Regulation and Target Therapy of Guangdong Higher Education Institutes, Sun Yat-sen University, Guangzhou, 510120 People’s Republic of China; 3 Department of Breast Surgery, Breast Tumor Center, Sun Yat-sen Memorial Hospital, Sun Yat-sen University, Yinfeng Road No. 33, HaiZhu District, Guangzhou, 510260 Guangdong People’s Republic of China; 4 School of Pharmaceutical Sciences, Sun Yat-sen University, Guangzhou, 510006 People’s Republic of China

**Keywords:** Contrast-enhanced ultrasound, Herceptin, Nanobubble, Phospholipid, Breast cancer

## Abstract

**Background:**

There is an unmet need for specific and sensitive imaging techniques to assess the efficacy of breast cancer therapy, particularly Her-2-expressing cancers. Ultrasonic microbubbles are being developed for use as diagnostic and therapeutic tools. However, nanobubbles circulate longer, are smaller, and diffuse into extravascular tissue to specifically bind target molecules. Here, we characterize a novel Herceptin-conjugated nanobubble for use against Her-2-expressing tumors.

**Methods:**

Phospholipid-shelled nanobubbles conjugated with Herceptin (NBs-Her) were fabricated using a thin-film hydration method and characterized in vitro in breast cancer cell lines and in vivo in a mouse model.

**Results:**

The average size of the unconjugated nanobubbles (NBs-Blank) and NBs-Her was 447.1 ± 18.4 and 613.0 ± 25.4 nm, respectively. In cell culture, the NBs-Her adhered to Her-2-positive cells significantly better than to Her-2-negative cells (*p* < 0.05). In vivo, the peak intensity and the half-time to washout of the NBs-Her were significantly greater than those of the NBs-Blank (*p* < 0.05). In addition, contrast-enhanced ultrasound imaging quality was improved through the use of the NBs-Her. The nanobubbles were able to penetrate into tumor tissue to allow extravascular imaging, but did not penetrate normal skeletal muscle.

**Conclusions:**

The Herceptin-conjugated nanobubble had many properties that made it useful for in vivo imaging, including longer circulation time and better tumor selectivity. This platform may be able to provide targeted delivery of therapeutic drugs or genes.

## Introduction

Approximately, 20–30 % of breast cancer patients are diagnosed with Her-2-positive breast cancers, which are associated with resistance to some chemotherapeutic agents and poor disease-free survival [1–3]. Early diagnosis is critical for successful treatment and improved prognosis of Her-2-positive breast cancer. Cytotoxic chemotherapy remains the mainstay treatment for breast cancer [4, 5]. More recently, neoadjuvant chemotherapy was established for breast cancer. These treatments aim to increase tumor respectability and allow breast-conserving therapies [6]. The combination of trastuzumab (also known as Herceptin), a humanized monoclonal antibody (mAb) against the Her-2 receptor [7], and neoadjuvant chemotherapy has improved the response rate for breast cancers that overexpress Her-2.

Recent studies have highlighted the importance of identifying patients who are not responsive to neoadjuvant chemotherapy early, so that subsequent treatment and postoperative chemotherapy regimens [8] can be altered to minimize toxicity and optimize the timing of surgery. Therefore, a sensitive and specific method to identify the tumor response to neoadjuvant chemotherapy is required. Contrast-enhanced ultrasonography (CEUS) is a useful diagnostic tool to detect the vascular pattern of breast cancers. It is more sensitive than conventional imaging modalities, such as mammography and B-mode ultrasonography, which is more susceptible to confounding effects from breast edema and fibrosis, common side effects of chemotherapy [9]. Ultrasonic molecular imaging could be a new diagnostic technique for estimating the efficacy of neoadjuvant chemotherapy in Her-2-positive breast cancers. However, in practice, the ultrasound microbubbles (MBs) currently used for CEUS are limited by their diameter (1–10 μm), can only produce blood pool contrast and are rapidly cleared from the cancer tissue, resulting in short-acting contrast. In addition, ultrasound MBs cannot be targeted to specific tumor antigens using antibodies or ligands on the bead surface, because they are unable to efficiently pass through the submucosal layers into tumor tissue.

Significant energy has been invested to develop nanotechnology tools for cancer diagnosis and therapy. Ultrasound contrast agents using this principle could be considered “theranostic,” combining therapy and diagnostics. They also have the advantage of being naturally acoustically activated. Unlike MBs, nanobubbles (NBs) efficiently penetrate through submucosal layers and have longer circulation times in vivo [10–13]. Rapoport et al. [14] confirmed that nanoscale particles can penetrate the tumor neovasculature and tumor tissue space, to achieve relatively long-acting contrast enhancement. NBs are also amenable to surface modifications that enhance their signal and tumor selectivity, and reduce non-specific toxicity. Targeting NBs through surface modifications is considered a promising theranostic approach. In preclinical medical imaging studies, Herceptin and its analogs have been shown to be effective targeting tools [15–19]. Thus, breast cancers that overexpress Her-2 are an attractive target for continued development of specific theranostic approaches. Moreover, equipping NBs with specific antibodies may be a simple means of producing specific targeted delivery systems [20].

In 2007, Liu et al. [20] reported the developments of polylactic acid (PLA) NBs conjugated with Herceptin, which showed specific imaging in vitro, but the NBs were not tested in vivo. Here, we developed a novel Herceptin-PEGylated phospholipid-shell NB reagent and characterized its in vitro and in vivo properties. We then evaluated the specificity, cytotoxicity, and efficiency of the NBs against Her-2-positive breast cancer cells.

## Materials and methods

### Preparing the nanobubbles

Bubbles were prepared using a thin-film hydration and sonication method [21, 22]. Briefly, a homogenous mixture containing 5 mg of 1,2-dipalmitoyl-sn-glycero-3-phosphoethanolamine (DPPE, Sigma-Aldrich, St. Louis, Missouri), 15 mg of 1,2-distearoyl-sn-glycero-3-phosphocholine (DSPC, Sigma-Aldrich, St. Louis, Missouri), 6.3 mg of polyethylene glycol (PEG4000, Aladdin Limited Company, Shanghai, China), and 2.2 mg of 2-distearoyl-sn-glycero-3-phosphoethanolamine-*N*-carboxy polyethylene glycol 2000 (DSPE-PEG2000-COOH, Avanti Polar Lipids Inc., Alabaster, Alabama) was made in 4 mL chloroform. The mixture was stirred for 2 h and then vacuum dried for 1 h at 60 °C using a rotary evaporator (EYELA, Tokyo, Japan). The resulting film was rehydrated with phosphate-buffered saline (PBS) to a concentration of 5 mg/mL and agitated at 50 °C at 120 rpm for 1 h.

The size of the resulting liposomes was reduced by sonication using a 100-kHz probe (Modal 220A, Branson Ultrasonics, Danbury) at high power (10 min, 120 W). The liposomal suspension was transferred to a tube and 5 mL of octafluoropropane (C_3_F_8_; Guangzhou Walter, China) gas was injected to replace the air over the fluid. Bubbles were created with additional sonication (30 s, 100 W). The initial bubbles ranged in size from 100 to 5000 nm and were purified to bubbles between 200 and 700 nm. First, the visible bubbles were discarded with a syringe, and a low-speed centrifugation (1000 rpm, 5 min) was performed to separate a thin upper layer in the suspension to discard large bubbles. Then the suspension was transferred to a 5 mL syringe and a higher centrifugation speed (2500 rpm, 15 min) was used. The bottom layer of liquid containing phospholipid fragments and liposomes was removed; then the NBs were collected and washed twice in PBS. Finally, the PEGylated nanobubbles were resuspended in 4 mL PBS and stored at 4 °C.

DiO (Beyotime, Haimen, China)-encapsulated bubbles were prepared using the same procedure, with the addition of DiO in the initial mixture of phospholipids and PEG in chloroform.

### Preparing Herceptin-targeted nanobubbles

Herceptin molecules were covalently bound to the PEGylated NBs (NBs-Blank) by linking the free amino groups of Herceptin and the carboxyl groups of DSPE-PEG2000 on the NBs. Briefly, 1-ethyl-3-(dimethylaminopropyl)carbodiimide hydrochloride (EDC, Sigma-Aldrich, St. Louis, Missouri) was mixed with *N*-hydroxysuccinamide (NHS, Sigma-Aldrich, St. Louis, Missouri) using an EDC:NHS:DSPE-PEG2000 molar ratio of 30:30:3 in a 2-(4-morpholino)ethanesulfonic acid (MES, Sigma-Aldrich, St. Louis, Missouri) solution (pH 5.5) for 30 min at room temperature. Then, the suspension was removed and centrifuged (1000 rpm, 5 min) three times to remove excess EDC and NHS. Herceptin (Hoffman La Roche, 1 mg/mL) was then added with a Herceptin/DSPE-PEG2000 molar ratio of 1:30 and incubated at 4 °C for 8 h (NBs-Her). Finally, the upper layer of the suspension was collected and washed (1000 rpm, 5 min) three times to remove the excess free Herceptin and stored at 4 °C.

### Determining bubble size and zeta potential

Bubble size and size distribution were measured at 25 °C using photon correlation spectroscopy at a scattering angle of 90° using a Zetasizer Nano ZS90 (Malvern Instruments, Worcestershire, UK). Samples were diluted 250-fold in distilled water to obtain the appropriate viscosity.

Zeta potential values were also obtained using a Zeta sizer NanoZS90 with an He–Ne laser beam at 633 nm and a scattering angle of 90° at 25 °C. The samples were dispersed in distilled water and the zeta potentials were calculated from the mean electrophoresis mobility using the Smoluchowski equation.

### Determining nanobubble concentration

The NBs concentration was determined using a hemocytometer. An NB sample fluorescently labeled with DiO was transferred to the hemocytometer and observed using a CarlZeiss Aviox-1 inverted fluorescence microscope (Carl Zeiss, Oberkirchen, Germany). A fluorescent and bright field image (400×) was obtained for each field of view (*n* = 3). The number of bubbles in each field was counted using the WCIF Image J software (v1.37; National Institutes of Health, Bethesda, MA). Sample concentrations (number/mL) were determined using the same method. All measurements were performed in triplicate.

### Western blot analysis

SDS-PAGE and Western blot were used to confirm whether Herceptin was bound to the NBs. An 8 % SDS–polyacrylamide gel was loaded with NBs-Blank, NBs-Her, and Herceptin and electrophoresed under reducing condition for 2 h at 60 mV and for an additional 180 min at 300 mA. The gel was then transferred to a membrane and blocked using 5 % skim milk. After blocking, the membranes were incubated overnight at 4 °C with a mouse antibody against ErbB2 (1:1000 dilution; Abcam, Cambridge, MA). Horseradish peroxidase (HRP)-conjugated donkey anti-mouse IgG (1:2000 dilution; Santa Cruz Biotechnology, Santa Cruz, CA) was used as the secondary antibody. Protein signals were detected using a chemiluminescence system (New Life Science Products, Boston, MA, USA).

### Cytotoxicity analysis

SK-BR3 (high Her-2 expression) and MDA-MB-231 (low Her-2 expression) breast cancer cells were provided by American Type Culture Collection (ATCC) [23]. The MDA-MB-231 cells were cultured in Dulbecco’s modified Eagle’s medium (DMEM, GIBCO Gaithersburg, MD, USA) with high glucose supplemented with 10 % fetal bovine serum and at 37 °C with 5 % CO_2_. The SK-BR3 cells were cultured in RPMI 1640 (GIBCO Gaithersburg, MD) with 10 % fetal bovine serum and at 37 °C with 5 % CO_2_. SK-BR3 and MDA-MB-231 cells were inoculated into 96-well plates at 2 × 10^3^ cells/well in 0.1 mL of medium for 12 h. The cells were then incubated for an additional 24 h in the same volume of fresh media with targeted or nonspecific NBs at 0, 2 × 10^4^, 2 × 10^5^, 2 × 10^6^, and 2 × 10^7^ bubbles/mL. After 24 h, the medium in each well was replaced with 100 μL of fresh medium containing 10 μL 3-(4,5-dimethylthiazol-2-yl)-2,5-diphenyltetrazolium bromide (MTT; 5 mg/mL) and incubated for an additional 4 h. Then, 100 μL of DMSO was added to dissolve the substrate after the MTT-containing supernatant was discarded. The plates were gently agitated for 5 min, and the absorbance of each well was determined at 540 nm using an Infinite F200 multimode plate reader (Spectra Max M5, Molecular Devices). All experiments were conducted in triplicate. The cell viability was calculated.

### Cell attachment studies

SK-BR3 and MDA-MB-231 cells were grown on chambered coverslips for 24 h. The plates were first washed three times with PBS to remove the dead cells. The cells were counterstained with 4′6-diamidino-2-phenylindole (DAPI, Beyotime, Haimen, China). The cells were incubated with approximately 5 × 10^5^ DiO-encapsulated NBs-Her or NBs-Blank for 30 min at room temperature, then washed three times with PBS to remove the unbound NBs. PBS (0.5 mL) was added to each well to differentiate between bound and unbound NBs, which floated to the surface. Images were captured with confocal laser scanning microscopy (CLSM, Zessi LSM 710, Germany). DiO and DAPI were excited at 484 and 364 nm, and emission was recorded at 501 and 454 nm, respectively.

To evaluate binding efficiency, cells were cultured on coverslips in 6-well plates until they reached 70 % confluence. The plates were washed twice with PBS, and 5 × 10^7^ DiO-encapsulated NBs-Her or NBs-Blank were added. Because the NBs are buoyant, the coverslips were inverted to facilitate contact between the cells and bubbles at room temperature. After 30 min, the coverslips were washed with PBS to remove unbound NBs. Finally, the cells were washed to the bottom of the plate and the supernatant was removed by centrifugation. The fluorescence intensity in the SK-BR-3 and MDA-MB-231 cells was analyzed by flow cytometry (Beckman Coulter, Fullerton, CA).

### In vivo tumor model and contrast-enhanced imaging

All animal experiments were carried out in accordance with the procedures and guidelines of the Institutional Animal Care and Use Committee and were approved by the Animal Experiment Committee and Biosafety Committee at Sun Yat-Sen University of Medical Science. Approximately, 3 × 10^6^ MDA-MB-231 or 6–8 × 10^6^ SK-BR3 cells were inoculated subcutaneously in the second fat pad of female nude mice (*n* = 8/group), half of which received NBs-Her and the other half NBs-Blank. The tumors were allowed to grow for 2 weeks before ultrasound imaging or tissue harvest. The mean maximum tumor size at ultrasound ranged from 5 to 9 mm. Mice were anesthetized with 2 % chloral hydrate by intraperitoneal injection and placed on a warm pad. The ultrasound contrast parameters were: probe frequency, 5–12 MHz; mechanical index (MI), 0.1; and gain 85 %. (PHILIPS, IU22 MATRIX). A B-mode image of the tumor was obtained first and used as an anatomical reference for quantification. Approximately, 1 × 10^8^ NBs-Blank (*n* = 4/group) or 1 × 10^8^ NBs-Her (*n* = 4/group) were injected through caudal veins in MDA-MB-231 or SK-BR3 tumor-bearing mice. Ultrasound contrast data were quantified with PHILIPS QLab8.1 software.

### Confirming NB tumor penetration

CLSM was used to confirm that the NBs passed through the inter-endothelial gaps in the tumors. The location of green-fluorescently dyed NBs was determined in vivo. SK-BR3 tumor-bearing mice were randomly separated into two groups to receive either NBs-Her or NBs-Bank. Approximately, 1 × 10^8^ DiO-labeled NBs were injected into the caudal veins. To clear the labeled bubbles from circulation, the heart was perfused with 0.9 % normal saline 3 h after bubble injection. The tumors and muscles of the right thigh (used as negative controls) were immediately extracted for sectioning into 5-μm slices. Frozen sections were stained in a solution of 2 μg/mL DAPI for 10 min to mark the nucleus. Images were recorded using laser scanning confocal microscope (LSCM, Zessi LSM 710, Germany).

### Statistical analysis

Count data were expressed as mean ± standard deviation. Data were compared using paired-sample *t* tests or univariate analysis of variance as appropriate. All statistical analyses were performed using SPSS software (Version 19; SPSS Inc. Chicago, IL, USA). *P* < 0.05 was considered to be significant.

## Results

### Characterization of the nanobubbles

The physical properties of the NBs are summarized in Table 1. The average diameter of NBs-Blank and NBs-Her was (447.1 ± 18.4) nm and (613.0 ± 25.4) nm, respectively. Zeta potential measurements showed that NBs-Blank had a net negative charge of −38.36 ± 0.81 mV (*n* = 5), while the charge of NBs-Her was −30 ± 0.42 mV (*n* = 5). The concentrations of NBs-Blank and NBs-Her were (1.22 ± 0.16) × 10^9^ bubbles/mL (*n* = 5) and (5.64 ± 0.19) × 10^8^ bubbles/mL (*n* = 5), respectively.

**Table 1:** Physicochemical characteristics of NBs

Formation	Size (nm)	PDI	Zeta potential (mV)	Concentration (/ml)
NBs	447.1 ± 18.4	0.211 ± 0.022	−38.36 ± 0.81	(1.22 ± 0.16) × 10^9^
Targeted NBs	613.0 ± 25.4	0.241 ± 0.045	−30.02 ± 0.42	(5.64 ± 0.19) × 10^8^

Data represent mean ± SD (*n* = 5)

*NBs* nanobubbles, *PDI* particle dispersion index

### Herceptin-binding efficiency to the NBs

**Fig. 1:**
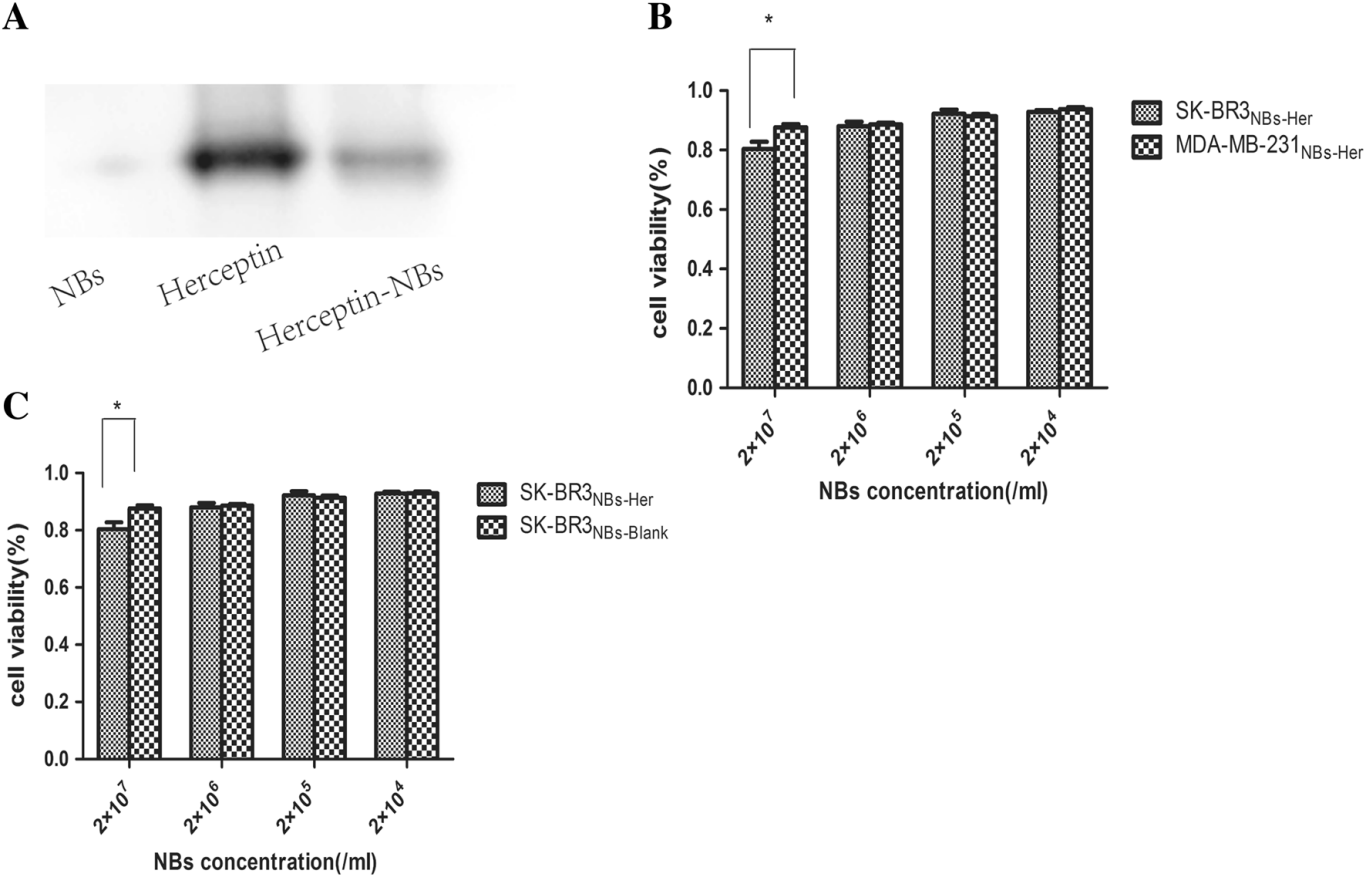
In vitro characterization of the Herceptin-conjugated nanobubbles. **a** Western blot analysis of the Herceptin-conjugated nanobubbles (NBs-Her) using an 8 % native polyacrylamide gel. *Lane 1* unconjugated nanonbubbles (NBs-Blank); *lane 2* Herceptin; *lane 3* NBs-Her. **b** SK-BR-3 and MDA-MB-231 cells were incubated with NBs-Blank for 24 and 48 h. There were no significant differences in the viability of SK-BR-3 or MDA-MB-231 cells cultured with NBs-Blank. **c** In vitro cytotoxicity assays using Her-2 positive SK-BR-3 cells (high Her-2 expression) and MDA-MB-231 cells (Her-2 negative) incubated with NBs-Her for 24 h. The viability of SK-BR-3 cells is significantly reduced by exposure to NBs-Her at high concentrations (*p* < 0.05). The *stars* indicate significant differences (*p* < 0.05). Cell viability data are expressed as mean ± SD (*n* = 3)

Western blot showed that the NBs-Her band was at approximately the same position as Herceptin (Fig. 1a: lane 3 versus 2), indicating that Herceptin was conjugated with the PEGylated NBs. Additionally, approximately 53.6 ± 0.43 μg of Herceptin was detected per 1 × 10^8^ NBs using a BCA protein assay. The deduced coupling efficiency is approximately 50 %, demonstrating that Herceptin was efficiently conjugated to the PEGylated NBs.

### Assessing the cytotoxicity of NBs against tumor cells

The cytotoxicity of NBs-Blank and NBs-Her was evaluated using SK-BR3 and MDA-MB-231 cancer cells incubated with NBs at four concentrations between 10^7^ and 10^4^/mL for 24 h (Fig. 1b, c). For the SK-BR-3 cells incubated with NBs-Blank incubated at 2 × 10^7^, 2 × 10^6^, 2 × 10^5^, and 2 × 10^4^/mL concentration after 24 h, the cell viability was measured to be 87.62 ± 2.59, 88.62 ± 1.49, 91.31 ± 1.91, and 92.36 ± 0.38 %. There were no significant differences in the viability of SK-BR3 cells cultured with NBs-Blank. The SK-BR-3 viability with the NBs-Her was 80.41 ± 2.91, 88.04 ± 3.38, 92.24 ± 1.16, and 92.89 ± 1.08 %. At higher concentrations (2 × 10^7^ NBs-Her/mL), the mortality rate of the SK-BR-3 cells was significantly increased (*p* < 0.05). However, given that the NBs-Blank was not toxic, the cytotoxicity was likely due to the antitumor effect of Herceptin. Similarly, 88.63 ± 1.03, 91.72 ± 4.74, 93.82 ± 7.75, and 92.23 ± 1.67 % MDA-MB-231 cells incubated with NBs-Her were viable. The cell viability of MDA-MB-231 cells incubated with NBs-Blank was 91.64 ± 4.51, 91.11 ± 1.01, 91.66 ± 0.91, and 93.21 ± 1.26 %, respectively. There were no significant differences in the viability of MDA-MB-231 cells cultured with NBs-Her or NBs-Blank. The cell viability of both SK-BR3 and MDA-MB-231 cells remained >80 % after incubation with either type of NBs, indicating they were minimally cytotoxic.

### Attachment of NB to tumor cells

**Fig. 2:**
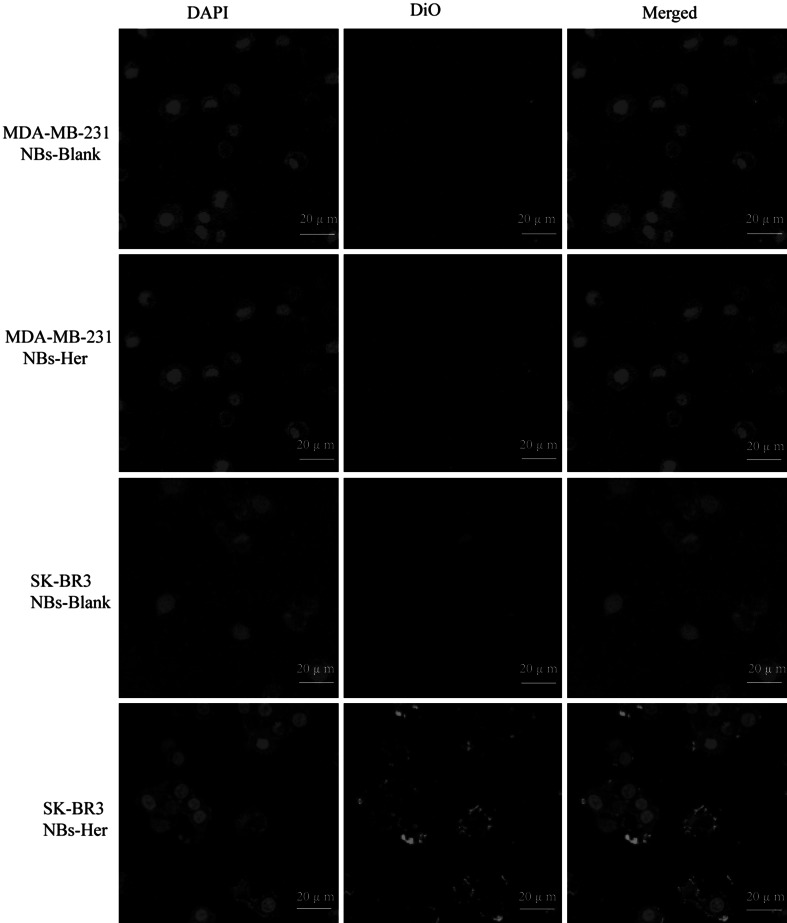
Nanobubbles adherence to tumor cells in vitro. The same quantity of Herceptin-conjugated nanobubbles (NBs-Her) and unconjugated nanobubbles (NBs-Blank) were added to SK-BR3 and MDA-MB-231 breast cancer cells and then observed using confocal laser scanning microscopy. **a** SK-BR-3 cells with NBs-Blank, **b** MDA-MB-231 cells with NBs-Her, **c** MDA-MB-231 with NBs-Blank, and **d** SK-BR-3 cells with NBs-Her. NBs-Blank did not adhere to either SK-BR3 or MDA-MB-231 cells (**a**, **c**), NBs-Her adhered to the SK-BR3 cells (**d**), but not MDA-MB-231 cells (**b**) (×200)

**Fig. 3:**
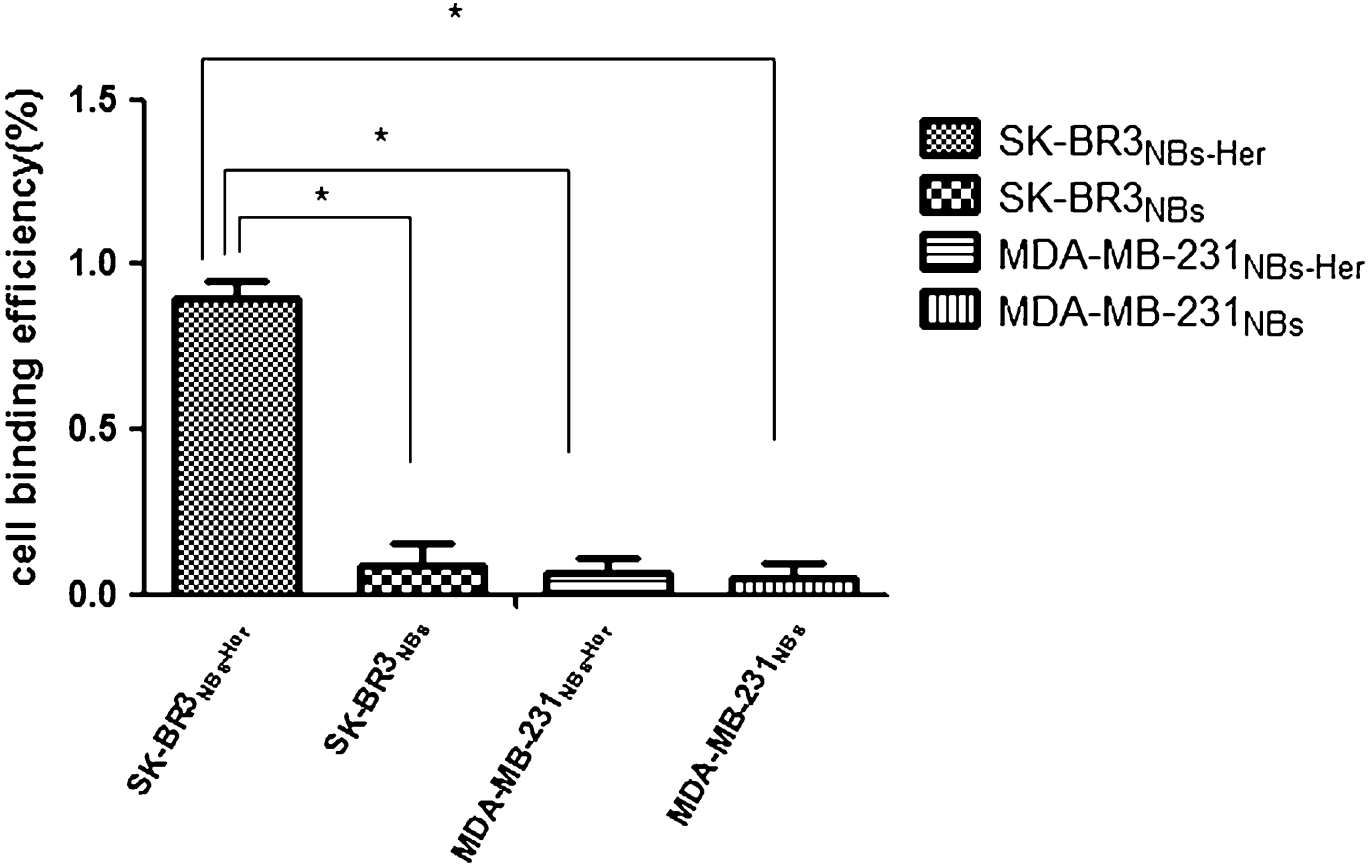
Assessing the binding efficiency of nanobubbles to tumor cells by flow cytometry. The efficiency with which Herceptin-conjugated nanobubbles (NBs-Her) or unconjugated nanobubbles (NBs-Blank) bound to MDA-MB-231 (Her-2 negative) and SK-BR-3 (Her-2 positive) breast cancer cells was assessed. The percentage of cells binding to the NBs is shown. The adherence of the NBs-Her was significantly higher in SK-BR-3 cells than the MDA-MB-231 cells (*p < 0.05). Data are reported as mean ± SD (*n* = 3)

Microscopically, the NBs-Her and NBs-Blank bubbles interacted with the SK-BR-3 and MDA-MB-231 cells in very different ways. There were most targeted NBs bound to the SK-BR3 cell membrane than the other three groups (Fig. 2). Flow cytometric analysis showed that the NBs-Blank did not attach to the SK-BR-3 and MDA-MB-231 cells (Fig. 3). NBs-Her also adhered to MDA-MB-231 cells at very low levels, but adhered at significantly higher levels to the SK-BR-3 cells (Fig. 3). In fact, the NBs-Her adhered to SK-BR3 cells approximately 10 times better than the NBs-Blank (*p* < 0.05). We did not observe any internalization of the NBs by the tumor cells.

### Stability and ultrasound sensitivity of the targeted NBs in vivo

**Fig. 4:**
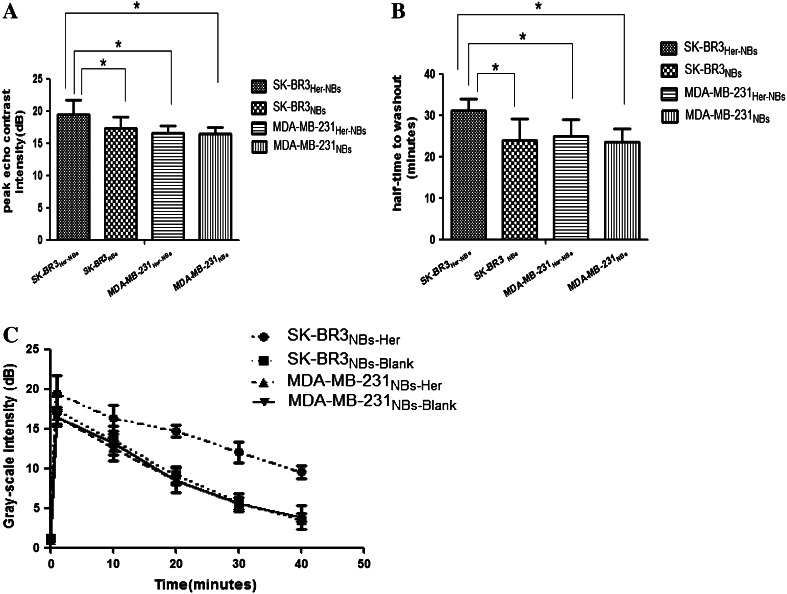
Time–intensity curve of contrast enhancement in tumors after injection of unconjugated (NBs-Blank) or Herceptin conjugated nanobubbles (NBs-Her). **a** Contrast agent images were acquired at peak intensity in mice with SK-BR-3 or MDA-MB-231 tumors (*n* = 4/group). **b**, **c** The peak intensity and half-time to washout of NBs-Her is shown. Both were obviously greater in mice with SK-BR-3 tumors treated with NBs-Her than SK-BR3-bearing mice injected with NBs-Blank or MDA-MB-231-bearing mice injected with NBs-Her (*p < 0.05). Data are shown in Table 2

The NBs-Her were tested in vivo in tumor-bearing mice that had been inoculated with either MDA-MB-231 cells (*n* = 8) or SK-BR-3 cells (*n* = 8). In the 16 mice from both groups, under the same ultrasound conditions, NBs-Her did not result in any recognizable symptoms of toxicity, and none of the animals exhibited apparent signs of distress after examination. The peak intensity, half-life of washout, and duration of contrast enhancement were compared between NBs-Blank and NBs-Her in MDA-MB-231 and SK-BR-3 tumors (Table 2). In the transplanted MDA-MB-231 tumors (low Her-2 expression), the peak intensity of NBs-Her (Fig. 4a) and NBs-Blank (Fig. 4a) was not significantly different (*p* = 0.886). Similarly, there was no significant difference in the half-time to washout between NBs-Her and NBs-Blank in the MDA-MB-231 model (Fig. 4b, *p* = 0.578). In contrast, in the transplanted SK-BR-3 tumors (high Her-2 expression), the peak intensity (Fig. 4a, *p* = 0.021) and the half-time to washout (Fig. 4b, *p* = 0.023) were significantly different between NBs-Her and NBs-Blank.

**Table 2:** Two indicators (mean ± SD) of blank and targeted NBs in two types of transplanted tumors

Tumor	Bubble	PI t (dB)	HT (min)
SK-BR3	Targeted NBs	19.46 ± 2.29	31.09 ± 2.85
	Blank NBs	17.37 ± 1.74	24.02 ± 5.03
MDA-MB-231	Targeted NBs	16.53 ± 1.21	25.09 ± 3.87
	blank NBs	16.42 ± 1.01	23.45 ± 3.26

*PI* peak intensity, *HT* half-time to washout

**Fig. 5:**
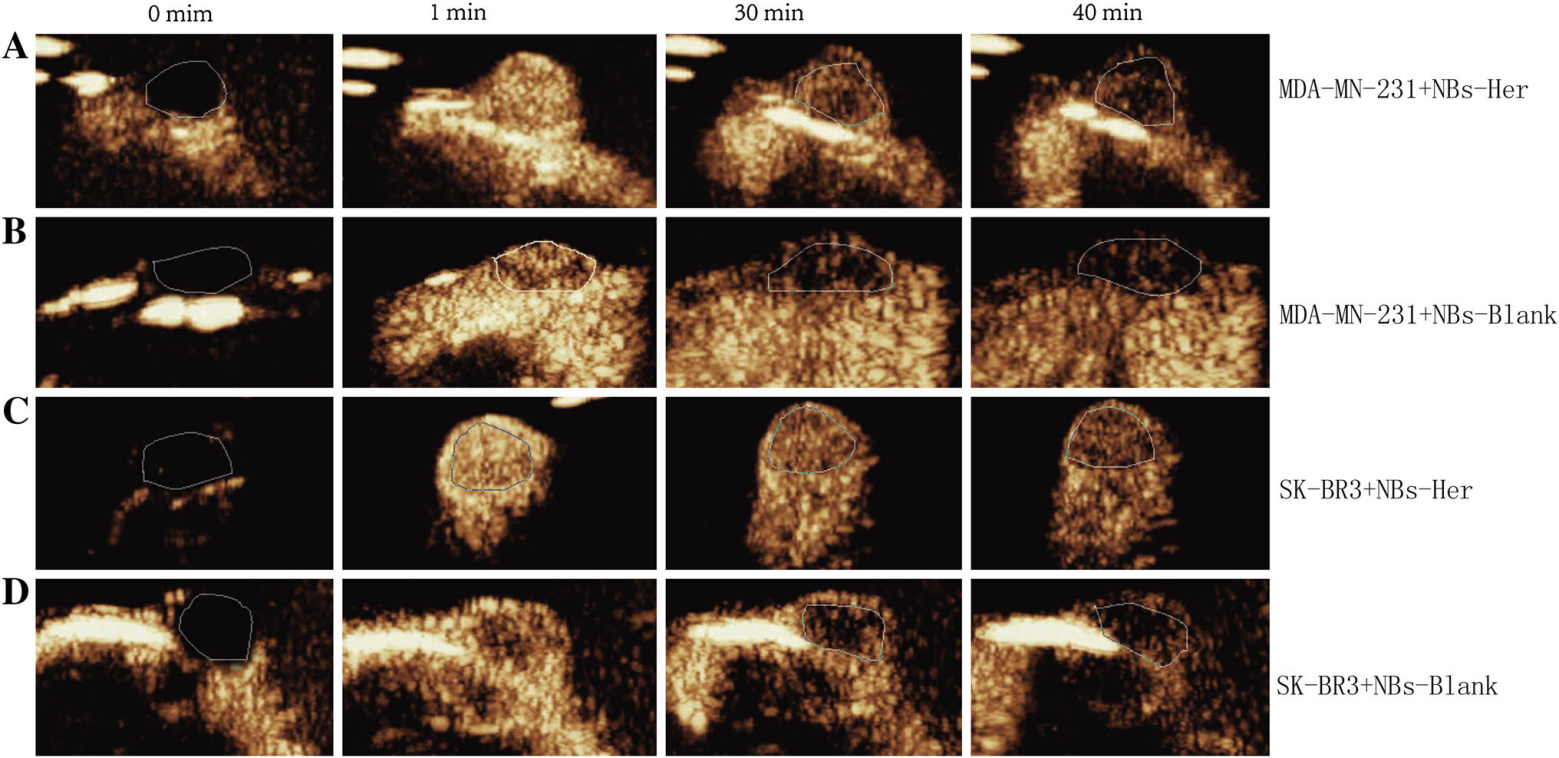
In vivo tumor targeting. Images were taken at the indicated time points (0, 1, 30, and 40 min) after nanobubbles were injected into the transplanted SK-BR-3 tumors injected with Herceptin-conjugated nanobubbles (NBs-Her; **a**) or unconjugated nanobubbles (NBs-Blank; **b)** and in the transplanted MDA-MB-231 tumors injected with NBs-Her (**c**) or NBs-Blank (**d**)

Contrast-enhanced images of the tumors continuously exposed to ultrasound were taken at 0, 1, 30, and 40 min (Fig. 5a–d). As shown in Fig. 5c, even after 40 min, the NBs-Her reagent still efficiently enhanced the contrast in transplanted SK-BR3 tumors, implying it has a longer duration of action in vivo in the SK-BR-3 tumors than in the other groups. The mice with SK-BR-3 tumors had a significantly higher peak intensity (*p* = 0.03) and half-time to washout (*p* = 0.045) than the mice with MDA-MB-231 tumors when they were treated with NBs-Her (Fig. 4a, b).

### Assessing NB tumor penetration

**Fig. 6:**
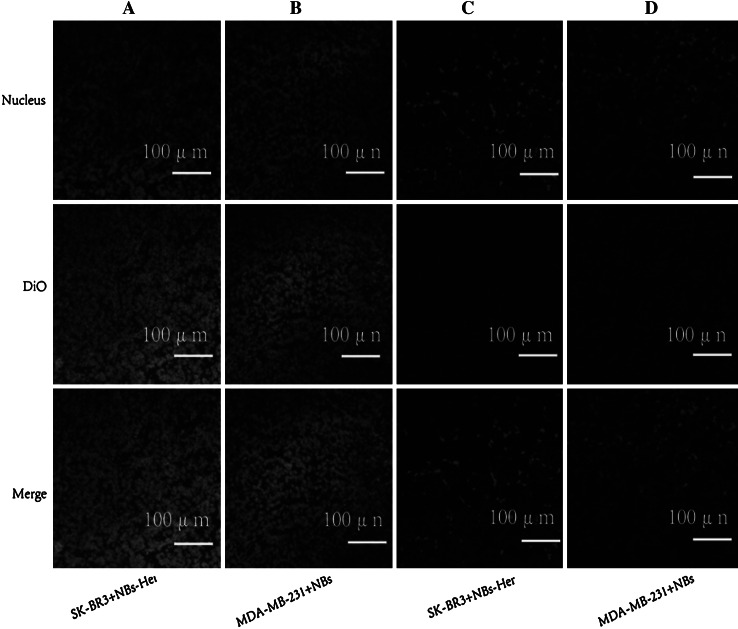
Tumor penetration by nanobubbles. Representative confocal laser scanning microscopy images of frozen sections after nuclear labeling are shown. A large number of DiO-labeled Herceptin-conjugated and unconjugated nanobubbles were observed in the tumor intercellular space (**a**, **b**), but DiO-labeled targeted and blank nanobubbles were difficult to detect in skeletal muscle (**c**, **d**)

Penetration of the NBs into the tumor was assessed using CLSM. The distribution of the DiO-labeled NBs-Her and NBs-Blanks were assessed in frozen sections of tumor and skeletal muscle (Fig. 6). In the transplanted MDA-MB-231 and SK-BR3 tumors, a considerable number of DiO-labeled NBs-Her (Fig. 6a) or NBs-Blank (Fig. 6b) were present in the intercellular space. In contrast, there were very few NBs in the skeletal muscle sections (Fig. 6c, d).

## Discussion

Herceptin has proven to be a useful reagent for both diagnostic and therapeutic strategies targeting Her-2 expression breast cancers [23]. Here, we have developed a novel Herceptin-conjugated NBs that is a putative theranostic strategy. The NBs-Her provide long-acting contrast enhancement, are non-toxic in vitro and in vivo, and specifically bind to tumor cells in vitro. Importantly, they also efficiently penetrate tumor tissue in vivo and are retained longer in tumors with high Her-2 expression, suggesting an interaction between the NBs-Her and Her-2.

One key innovation in the NBs-Her reagent is the use of DSPE-PEG2000-COOH as a linker lipid rather than the traditional streptavidin/biotin conjugation method [24]. The streptavidin/biotin system has not been widely used for clinical applications, because biotin–protein bonds may lead to immunogenicity [25–28]. Instead, we used carbodiimide-mediated chemistry to target the NBs using the DSPE-PEG2000-COOH linker. The binding rate between Herceptin and the NBs was efficient. In vitro, a significantly larger number of mAb-modified NBs-Her adhered to the SK-BR3 (high Her-2) cells than to the MDA-MB-231 cells (low Her-2), suggesting that the adhesion of NBs to Her-2-positive breast cancer cells is due to the antigen–antibody reaction. This confirmed that NBs-Her could be efficiently targeted. In vitro cytotoxicity testing also showed that NBs-Her and unconjugated NBs were non-toxic at concentrations up to 10^7^ bubbles/mL. Slightly increased cell mortality was observed in the SK-BR3 cells than the MDA-MB-231 cells, which is likely attributable to the antitumor activity of Herceptin. Therefore, the low cytotoxicity of NBs-Her suggests it could be a promising reagent for tumor imaging.

We tested the imaging capabilities of NBs-Her in vivo in mice inoculated with either SK-BR-3 or MDA-MB-231 tumor cells. Due to the negative correlation between bubble size and acoustic backscatter intensity, smaller gas-filled particles, like NBs, were predicted to be difficult to detect by ultrasound [29]. However, Yin et al. confirmed in a mouse model that NBs were able to produce strong contrast enhancement that persisted for more than an hour, while the enhanced contrast signal provided by MBs decayed significantly after 15 min. Similarly, we observed enhanced contrast for more than 40 min at an ultrasound frequency of 5–12 MHz, achieving optimal echogenicity intensities. This could be because the NBs passed through the endothelial gaps in the tumor in greater numbers, were retained in the tumor tissue for longer, and aggregated into micron-sized clusters in the extravascular space [14].

The parameters peak intensity and half-time to washout may be used as indicators of successful targeting for NB-enhanced imaging. In the SK-BR-3 model, which expressed high levels of Her-2, the peak intensity and half-time to washout of NBs-Her were significantly higher than NBs-Blank. However, in the MDA-MB-231 model, which had low levels of Her-2 expression, the peak intensity and half-time to washout were not significantly different between the two types of NBs. The extended retention time in the SK-BR-3 model is likely due to the increased number of interactions between antigen and antibody in the transplanted SK-BR3 tumors. Our results are similar to those published by Wang et al. [30].

CLSM imaging was used to determine if the NBs penetrated through inter-endothelial gaps in the tumors. The lack of a basement membrane and smooth muscle and the expansion of the intercellular space in cancer vasculature result in a maximum pore size of approximately 380–780 nm [28]. This is ample space for NBs with a diameter <700 nm to pass through the tumor neovasculature to allow extravascular imaging. CLSM imaging was used to investigate penetration and accumulation of DiO-labeled NBs in tumors. DiO labeled the tumors with green fluorescence for imaging. We did not observe significant differences in tumor penetration between NBs-Her and NBs-Blank in the SK-BR3-transplanted mice. This could be because multiple DiO-labeled NBs-Her or NBs-Blank penetrated the inter-endothelial gaps and were retained in the tumor for a long time more than an hour. Another possibility is that the fluorescence was quenched during the preparation of the frozen sections, while DiO-labeled NBs fluoresces green in tumor cells in vivo. We did observe a significant difference in the fluorescence of the tumors and the frozen thigh sections, likely due to the inability of NBs to pass through the inter-endothelial gaps of normal tissue.

Our study had some limitations. In vitro, the ability of NBs to attach to tumor cells was not assessed under flow conditions that would simulate in vivo exposure of the NBs to shear stress, which may impact the number of NBs attached to cells. In addition, future NB attachment studies in vitro can directly compare NB binding with Her-2 density by using cell lines with different levels of receptor expression. In vivo, our studies were limited by the relatively small number of animals.

## Conclusion

In conclusion, noninvasive and inexpensive sonography combined with Herceptin-targeted NBs may be a promising tool for diagnosing and evaluating the treatment response of Her-2-positive breast cancer. Furthermore, Herceptin-targeted NBs may also provide a targeted therapy platform if they are coated with chemotherapeutic drugs or genes. Therefore, the Herceptin-targeted NBs described here may be a multifunctional tool with promising clinical applications.
